# Tagatose consumption provokes metabolic syndrome features in rat males from mothers that consumed fructose during their pregnancy

**DOI:** 10.1186/s10020-025-01402-3

**Published:** 2025-12-29

**Authors:** Elena Fauste, Madelín Pérez-Armas, Cristina Donis, Paola Otero, Mª Isabel Panadero, Carlos Bocos

**Affiliations:** 1 Facultad de Farmacia, Universidad San Pablo-CEU, Urbanización Montepríncipe, Boadilla del Monte, Madrid, 28668 Spain; 2 Instituto Universitario de Estudios de Las Adicciones (IEA-CEU), Universidad San Pablo-CEU, CEU Universities, Montepríncipe, Boadilla del Monte, Madrid, 28668 Spain

**Keywords:** Fructose, Pregnancy, Foetal programming, Bile acids, FGF21, Angiotensin II, Tagatose

## Abstract

**Background:**

Maternal fructose intake induces harmful effects in progeny. However, this sugar is not contraindicated during pregnancy. On the other hand, the use of low-calorie sweeteners, such as tagatose, is increasing. Thus, we have studied whether the consumption of tagatose compared to fructose affects lipid metabolism in the offspring of mothers which were supplemented with fructose during their pregnancy.

**Methods:**

Three-month-old male rat offspring from control or fructose mothers received liquid 10% fructose or tagatose for 21 days. A control group (without any additive) was also included. Biochemical and molecular parameters were determined in plasma, tissues and feces.

**Results:**

Both tagatose and fructose consumption caused hypertriglyceridemia in descendants of fructose-fed mothers. Whereas fructose consumption led to a greater hepatic lipogenesis, tagatose supplementation provoked a higher enterohepatic bile acids recirculation, and therefore a higher intestinal lipid absorption and assembly. However, plasma GLP1, a molecule that affects lipid intestinal absorption, was unchanged. Curiously, FGF21, a molecule which regulates lipid and carbohydrate metabolism and is sensitive to GLP1, was augmented in plasma and liver of tagatose-supplemented descendants regardless of their maternal diet. Interestingly, Angiotensin II (Ang II), which can induce FGF21 production, was increased in plasma of all animals supplemented with tagatose. However, the deleterious effects of Ang II were effectively reversed by FGF21 in males from control mothers, but not in descendants of fructose-fed dams.

**Conclusions:**

Maternal fructose consumption determines the response of the offspring to tagatose intake, causing an increased intestinal lipid absorption, and metabolic changes that are characteristic of metabolic syndrome such as dyslipidaemia, steatosis and oxidative stress.

**Supplementary Information:**

The online version contains supplementary material available at 10.1186/s10020-025-01402-3.

## Introduction

In recent decades, metabolic diseases such as obesity, metabolic syndrome and diabetes have reached epidemic proportions in many countries (Carrera-Bastos et al. 2011; Kopp 2019). Various studies have shown how metabolic changes that occur during the pre- and postnatal development modulate the risk of developing these diseases, once adult. This phenomenon is called fetal programming and, among all the causal factors, nutrition during gestation is one of the most determinant parameters (Howie et al. 2009).

Fructose is a monosaccharide found in fruits, honey, sugar beets, and sugar cane. In recent years, there has been an increase in the consumption of added sugars, mainly fructose, in our diet, since they are widely used by the food industry as sweeteners (White and Nicklas 2016). In fact, fructose is present in a wide variety of processed foods such as industrial pastries, sauces and sugary drinks, which has led to a drastic increase in fructose consumption in the population. Several studies carried out both in experimental animal models and clinical studies in humans have shown that a high fructose intake contributes to the increased incidence of metabolic diseases (Alwahsh and Gebhardt 2017; Johnson et al. 2024). Nevertheless, the consumption of sugary drinks or foods that contain added fructose is not contraindicated during pregnancy.

Although in developing countries an increase in the use of added sugars has been found in recent years, in developed countries this trend has changed and the consumption of added sugars has stabilized or even decreased, possibly because social awareness policies are proving effective. Despite these data, the percentage of childhood and adult obesity has continued to increase in recent decades, but more slowly. Thus, in a study conducted by Faruque et al. (2019) in the United States, as an example of many other countries, they observed that the drastic increase (annual rate of change = + 1.33) in sugar consumption from the 1970 s to the 1990 s was followed in parallel by a subsequent exponential growth (+ 0.82 and + 0.97) in the prevalence of obesity that lasted until the 2000s. Interestingly, after the drop (−0.91) in sugar consumption that occurred from the 1990 s to the 2010 s, a slowdown in the annual increase (+ 0.37) in the prevalence of obesity has been observed from the 2000 s onwards (Faruque et al. 2019).

In our laboratory, we have developed an "animal model of fetal programming" induced by maternal fructose intake in which the characteristics of metabolic syndrome appear in the offspring naturally or induced after supplementation with liquid fructose. Thus, male offspring from mothers consuming fructose during pregnancy had characteristics of metabolic syndrome, such as hepatic steatosis and hyperinsulinemia; these effects were produced exclusively through epigenetic mechanisms caused by a fetal programming mechanism. Although females from fructose-fed mothers did not appear to be affected by the maternal diet, when they received liquid fructose once adult, they showed an exaggerated response to fructose intake, characterized by hyperlipidaemia and fatty liver (Rodríguez et al. 2016a). With this and other findings, we could confirm that maternal fructose intake determines the response of the offspring to the diet.

These results found in experimental animals could explain why a drastic reduction in the consumption of added sugar has not been accompanied by a parallel decrease in the rate of obesity and various metabolic diseases, as observed by Faruque et al. in their study (Faruque et al. 2019). In fact, it is surprising the case of individuals that despite following a healthy diet, they develop metabolic diseases without any apparent reason. The explanation could be found in epigenetic changes derived from the fact that during pregnancy their mothers consumed large amounts of sugary drinks or processed foods. Related to that, a recently published article would support this hypothesis. Gracner et al. studied the exposure to sugars within 1000 days of conception and its impact on diabetes and hypertension. They focused on sugar consumption in the UK population before and after the end of sugar and sweets rationing in 1953. During rationing, sugar intake was at levels within the current recommended dietary guidelines. However, after the end of rationing, consumption almost doubled. Comparing adults conceived pre- or post-rationing, it was observed that the decrease in sugar consumption during the perinatal period reduced the risk of diabetes and hypertension by approximately 35% and 20%, respectively. Interestingly, intrauterine sugar rationing alone accounted for about one-third of the reduction in the risk of developing metabolic disease when adult (Gracner et al. 2024).

Therefore, carbohydrates with sweetening properties and a low caloric value are being investigated to be used as alternative sugars to fructose. Tagatose (an epimer of fructose) is a rare sugar that has antioxidant and prebiotic effects, reduced glycaemic and insulinemic responses, and the potential to improve lipid profile, to induce a lower expression of proinflammatory cytokines, catalase and superoxide dismutase, to decrease lesion area and macrophage infiltration, and to stimulate GLP1 release, therefore constituting an alternative candidate for the treatment of diabetes mellitus and obesity (Guerrero-Wyss et al. 2018; Nagata et al. 2018). In fact, tagatose reached a phase 3 clinical trial to determine its value as an antidiabetic agent.

Unlike fructose (4 kcal/g), tagatose is a low-calorie sugar since its energy value is estimated to be 1.5 kcal/g. Currently, tagatose is being added to soft drinks, cereals, chocolate, sweets, caramels, yogurts, ice creams, nutritional supplements and dairy products. Interestingly, only about 20% of consumed tagatose is absorbed by the small intestine, with most of it fermented by colonic bacteria into short-chain fatty acids (SCFA), which are almost completely absorbed. It is mainly metabolized by the liver in a similar way to fructose, with little tagatose reaching the systemic circulation. Tagatose has been recognized by the Food and Drug Administration (FDA) as GRAS (*Generally Recognized As Safe*) and approved as a “new food ingredient” by the European Union, without any restrictions on its use (Smith et al. 2022; Ortiz et al. 2024; Lu et al. 2008) for the general population, including pregnant women. In fact, studies on the effects of non-nutritive sweeteners such as tagatose have recently been piloted in pregnant women (López-Arana et al. 2023). Nevertheless, some studies have described that high doses of tagatose can cause transient gastrointestinal side effects, such as diarrhea, although they were usually mild. In addition, some studies concluded that foods containing tagatose would not be suitable for people with disorders in fructose metabolism, as tagatose is metabolised in the same way as fructose. In any case, tagatose represents an interesting sugar alternative compared to its isomer fructose, as it has a reduced deleterious impact on both the metabolic profile and the related cardiac susceptibility to ischemia–reperfusion injury (Durante et al. 2021). Tagatose could also be beneficial for metabolic health in women with insulin resistance characterized by hyperinsulinism because it reduces the glycaemic response and without stimulation of C peptide release (Sambra et al. 2022).

With these antecedents and considering that maternal fructose intake determines the response of the offspring to the diet and the increasing use of rare sugars as alternative to the fructose as added sweeteners, we investigated in the present work the effects of maternal fructose consumption on the response of the progeny to tagatose intake for 21 days, in comparison to that of fructose.

## Material and methods

### Animals and experimental design

An animal model of maternal liquid fructose intake was developed as previously described (Fauste et al. 2020; Rodrigo et al. 2018; Rodríguez et al. 2013). Female Sprague–Dawley rats weighing 200–240 g were fed ad libitum, a standard rat chow diet (Teklad Global 14% Protein Rodent Maintenance Diet, Envigo, USA), and housed under controlled light and temperature conditions (12-h light–dark cycle; 22 ± 1ºC). The experimental protocol was approved by the Ethical Committee for Animal Experimentation of the University San Pablo-CEU and by Autonomous Government of Madrid (ref. numbers 10/206458.9/13 and 10/042445.9/19).

Pregnant rats were randomly separated into a control group (no supplementary sugar) and a fructose-supplemented group (fructose 10% wt/vol in drinking water) (7–8 rats per group) throughout gestation (Rodríguez et al. 2013). Pregnant rats were allowed to deliver and on the day of birth, each suckling litter was reduced to nine pups per mother. After delivery, both mothers and their pups were maintained with water without any additives and food ad libitum. At 21 days of age, pups were separated by gender and males were fed a standard rat chow diet (Teklad Global 14% Protein Rodent Maintenance Diet, Envigo, USA) and water. The female progeny of each litter was used for a separate experiment. When the male offspring was 3 months old, they were subjected to a new dietary treatment for 21 days regardless of the group of mothers they were born. Male progeny from control or fructose-fed mothers were randomly separated into three experimental groups: control (C, tap water), fructose (F, fructose), and tagatose (T, tagatose), all sugars added as 10% wt/vol in drinking water. Animals within each experimental group were born to different dams to minimize the “litter effect” and the cages contained a maximum of four males to reduce distress. Intake of solid food and liquid per cage were daily recorded and the area under the curve (AUC) for the consumed chow, the ingested liquid and the total amount of ingested calories were calculated. After 21 days of dietary treatment, male offspring were killed. Before this, rats gradually lost consciousness with carbon monoxide. Food and liquid sugar were removed two hours before sacrifice. Blood was collected into EDTA-containing tubes, plasma was obtained by centrifugation and stored at −20ºC until processed. Liver, ileum, heart and lumbar adipose tissue, and the last two feces from the rectum were immediately removed, placed in liquid nitrogen, and kept at −80 ºC until analysis.

### Plasma determinations

Plasma aliquots were used to determine triglycerides and total bile acids (Spinreact, Girona, Spain) using commercial kits. GLP1 (Cusabio, Wuhan, RPC), FGF21 (R&D Systems, USA), copeptin (Cloud-Clone Corp., Wuhan, RPC) and Angiotensin-II (Cusabio, Wuhan, RPC) were determined in plasma samples using specific ELISA kits for rats.

### Tissue determinations

Two hundred milligrams of frozen tissue and one hundred milligrams of feces were immersed in chloroform:methanol 2:1 plus butylhydroxytoluene (BHT) (50 mg/L) and used for lipid extraction following the Folch method. Aliquots of lipid extracts were dried, and the remaining residue was weighed to determine total lipid content. Triglycerides were measured using the following procedure, briefly, 1 mL of Triton-X 100 1.25% in chloroform was added to 0.3 mL of lipid extracts, dried, and resuspended in 0.5 mL of distilled water. Triglycerides were measured using an enzymatic colorimetric assay (Spinreact, Girona, Spain).

Hepatic glycogen was extracted by using ethanol. Briefly, 100 mg of liver was degraded by using 30% KOH and boiling. After that, glycogen was precipitated with ice-cold 99% ethanol for 24 h and after centrifugation, the pellet containing glycogen was resuspended in distilled water. Glycogen was hydrolyzed to glucose monomers with an acidic hydrolysis and neutralized before glucose measurement with a colorimetric kit (Spinreact, Gerona, Spain).

One hundred milligrams of liver were homogenized in 1.2 mL PBS. After centrifugation, supernatants were used to measure bile acids (Spinreact, Girona, Spain). For bile acid measurement in feces, 0.2 g of feces were dried, and bile acid extraction was performed using 0.5 mL of methanol. After centrifugation, supernatants were used to measure bile acids (Spinreact, Girona, Spain).

One hundred milligrams of frozen tissue were homogenized in 0.25 M Tris–HCl, 0.2 M sucrose, and 5 mM dithiothreitol (DTT) buffer at pH 7.4 to determine the oxidative stress state. Thus, the concentration of malondialdehyde (MDA) was measured as a marker of lipid peroxidation using the method previously described (Wong et al. 1987), by measuring the fluorescence of MDA-thiobarbituric acid (TBA) complexes at 515 nm/553 nm excitation/emission wavelengths. Catalase activity was studied by the H_2_O_2_ disappearance caused by the activity of this enzyme (Aebi 1984) This was done by recording the absorbance maximum of H_2_O_2_ at 240 nm. Finally, the activity of superoxide dismutase (SOD) was measured using a commercial kit (Merck-Sigma, USA).

### RNA extraction and gene expression by qPCR

Total RNA was isolated from the tissues using Ribopure (Invitrogen, ThermoFisher Scientific, USA). Total RNA was subjected to DNase I treatment using Turbo DNA-free (Invitrogen, ThermoFisher Scientific, USA), and RNA integrity was confirmed by agarose gel electrophoresis. Afterwards, cDNA was synthesized by oligo(dT)-primed reverse transcription with Superscript II (Invitrogen, ThermoFisher Scientific, USA). qPCRs were performed using a CXF96® Touch (Bio-Rad, California, USA). The reaction solution was carried out in a volume of 20 μl, containing 10 pmol of both forward and reverse primers, 10 × SYBR Premix Ex Taq (Takara Bio Inc., Japan), and the appropriate nanograms of the cDNA stock solution. *Rps29* was used as a reference gene for qPCR. The primer sequences were designed using primer-BLAST software (NCBI) (Ye et al. 2012).

Samples were analysed in duplicate on each assay. Amplification of non-specific targets was discarded using the melting curve analysis method for each amplicon. qPCR efficiency and linearity were assessed by optimization of the standard curves for each target. The transcription was quantified with CFX Maestro 2.0 software (Bio-Rad, California, USA) using the efficiency correction method (Pfaffl 2001).

### Statistical analysis

Results were expressed as means ± S.E. Treatment effects were analyzed by two-way analysis of variance (ANOVA) with maternal diet (M) and progeny diet (D) as factors. Then, the Bonferroni test was used for post hoc analysis to identify the source of significant variance. Data that were not normally distributed were log-transformed to achieve data normality. Significant differences (*p* < 0.05) were indicated either with asterisks (*) between groups of animals receiving different nutritional treatments but belonging to the same dietary group of mothers or hash symbols (#) between groups of rats receiving the same treatment but coming from different dietary groups of mothers. The eta2 (η2) parameter (i.e., the proportion of the total variance that was attributed to the corresponding effect (M, D, or the M × D interaction)) was also provided (Table S1). All statistical analysis were performed using SPSS version 29 computer program.

## Results

### Food, liquid, and total caloric intake were not modified by tagatose consumption

**Fig. 1:**
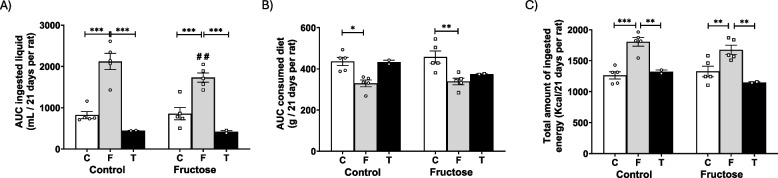
Food, liquid, and total caloric intake were not modified by tagatose consumption. **A** AUC ingested liquid, **B** AUC consumed chow, and **C** total amount of ingested energy from control (C, empty bar), fructose- (F, light grey bar), and tagatose-supplemented (T, dark grey bar) male progeny from control (left panel) or fructose-fed (right panel) mothers. Data are means ± S.E. from 7–8 litters. Asterisks denote a significant difference (*, *p* < 0.05; **, *p* < 0.01; ***, *p* < 0.001) between the groups under the crossbar (groups with a different diet but the same mother´s diet). Hash symbols denote a significant difference (#, *p* < 0.05; ##, *p* < 0.01; ###, *p* < 0.001) as compared to the control mothers (groups with the same diet but different mother´s diet). AUC: area under the curve

The sweetening power of tagatose is only slightly less than that of sucrose with a relative sweetness of 92% when compared in 10% solutions, and it is not so high as that of fructose, which is the sweetest of all naturally occurring carbohydrates (Aidoo et al. 2013) The relative sweetness of fructose has been reported in the range of 1.2–1.8 times that of sucrose. Curiously, whereas the intake of liquid was significantly increased in males consuming 10% fructose when compared to males that received water without additives, as previously published (Fauste et al. 2024), the AUC of ingested liquid trended to be lower in males receiving 10% tagatose versus their respective control males, although without reaching statistical significance in any case (Fig. 1A). These effects were observed regardless of their maternal diet.

Male offspring from control mothers consumed 47% of their total calories from fructose, while in males from fructose-fed mothers, around 41% of the total amount of energy was acquired from fructose. On the other hand, tagatose is considered to provide 1.5 kcal/g, due to its low absorption rate, compared to sucrose or fructose that provide 4.0 kcal/g (Roy et al. 2018), consequently, only 5% and 5.5% of total calories come from tagatose in the progeny from control and fructose-fed mothers, respectively. According to this, in both groups subjected to liquid fructose, solid diet consumption was similarly reduced to compensate for the calories obtained from fructose. However, in the case of tagatose, this compensatory effect for the calories ingested from sugar was not observed in descendants from control mothers but it was found in progeny from fructose-fed dams, although without being significantly different (Fig. 1B). Thus, in males from control mothers, the total amount of ingested energy was the same in descendants drinking water without additives as in males supplemented with tagatose (Fig. 1C). As previously observed by us (Rodríguez et al. 2016a), this compensatory effect turned out to be inefficient in the case of descendants consuming fructose.

Interestingly, these differences in the total calorie intake observed between the experimental groups, mainly in comparison to the animals that consumed fructose, were not reflected in the final body weight. There were no differences in the final body weight between the different dietetic experimental groups, neither in progeny from control mothers nor in descendants from fructose-fed dams (Table S2). And the same situation was found for the weights of diverse organs: liver, heart and lumbar adipose tissue. This situation was observed although the gain in body weight was, logically, lower in all the descendants that consumed tagatose (Table S2). It is noteworthy that differences were found between progeny from control and fructose-fed mothers (indicated by hash symbols). These modifications are due to the significantly lower body weight observed in all descendants of fructose-fed mothers before starting the dietary treatments, as already we have described in previous studies (Rodríguez et al. 2016a, 2016b).

### Tagatose induced ChREBP target genes in ileum but not in liver

**Fig. 2:**
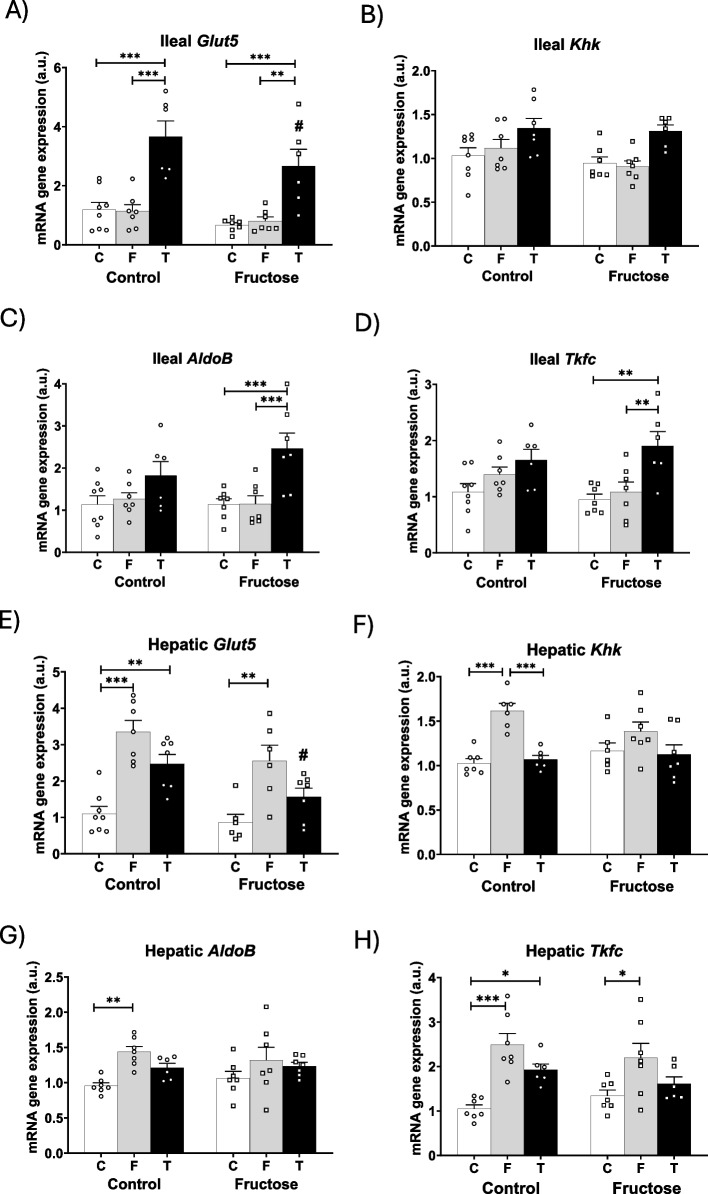
Tagatose induced ChREBP transcriptional activity in ileum but not in liver. **A***Glut5*, **B***Khk*, **C***Aldo B*, **D***Tkfc* mRNA ileal gene expression, and **E***Glut5*, **F***Khk*, **G***Aldo B*, **H***Tkfc* mRNA liver gene expression from control (C, empty bar), fructose- (F, light grey bar), and tagatose-supplemented (T, dark grey bar) young male progeny from control (left panel) or fructose-fed (right panel) mothers. Data are means ± S.E. from 7–8 litters. Relative target gene mRNA levels were measured by Real-Time PCR as explained in Materials and Methods, normalized to Rps29 levels and expressed in arbitrary units (a.u.). Asterisks denote a significant difference (*, *p* < 0.05; **, *p* < 0.01; ***, *p* < 0.001) between the groups under the crossbar (groups with a different diet but the same mother´s diet). Hash symbols denote a significant difference (#, *p* < 0.05; ##, *p* < 0.01; ###, *p* < 0.001) as compared to the control mothers (groups with the same diet but different mother´s diet). ChREBP: carbohydrate-responsive element-binding protein; *Glut*: glucose transporter; *Khk*: ketohexokinase; *Aldo*: aldolase; *Tkfc*: triokinase and FMN Cyclase

Given the different intake of tagatose versus fructose observed in all descendants, we were first interested in confirming whether these sugars were producing any effect on their own metabolism. It is known that ChREBP (carbohydrate responsive element binding protein) is a transcription factor that responds to sugar consumption (Katz et al. 2021). Moreover, ChREBP activity in vivo appears to be more responsive to sugars other than glucose and, in fact, it is potently activated by fructose ingestion. ChREBP co-ordinately regulates the expression of all three fructolytic enzymes: ketohexokinase (*Khk*), aldolase b (*AldoB*), and triokinase and FMN cyclase (*Tkfc*) (Herman and Birnbaum 2021). Interestingly, the metabolism of tagatose is identical to that of fructose (Guerrero-Wyss et al. 2018). Therefore, the expression of these enzymes and the specific transporter for the entry of these sugars (glucose transporter 5, *Glut5*) to the cells was determined both in the intestine (the first organ able to metabolize them) and the liver (which is supposed to be the main organ in charge of metabolizing them). Curiously, the effects observed were again quite different between the two carbohydrates. Unexpectedly, tagatose appeared to be metabolized more efficiently than fructose in the ileum, even though it had been ingested in significantly smaller amounts. Thus, tagatose (but not fructose) induced significantly *Glut5* gene expression in comparison to the other dietary treatments in all descendants, being more evident in descendants from control mothers (Fig. 2A). Moreover, tagatose (in contrast to fructose) induced the gene expression of the three tagatolytic enzymes in all descendants, although, in this case, the effect was more pronounced and significant in progeny from fructose-fed mothers (Fig. 2B-D).

Regarding the liver, the situation was different. Both sugar transport and its metabolism were preferentially induced by fructose and barely activated by tagatose. Thus, fructose activated *Glut5*, *Khk*, *AldoB*, and *Tkfc* hepatic gene expression (Fig. 2E-H) showing a similar profile than the intake of liquid fructose (Fig. 1A). Tagatose, however, induced *Glut5* and *Tkfc*, but not *Khk* and *AldoB*, being this effect only found in males from control mothers. Interestingly, tagatolysis was less induced in the intestine of these animals (Fig. 2A-D) than in progeny from fructose-fed dams and, therefore, some amount of tagatose is supposed to be able to reach the liver, leading to the effects observed in this tissue (Fig. 2E-H).

To corroborate these unexpected findings in the fructolytic pathway, the gene expression of lipogenic genes regulated by ChREBP (Herman and Birnbaum 2021), as well as its own gene expression, were determined in both ileum and liver. The results shown in Table S3 would confirm that tagatose induced ChREBP target genes in the ileum, whereas fructose did so in the liver. Thus, tagatose (but not fructose) induced ileal gene expression of *Chrebp* in all descendants compared to the other dietary treatments, becoming significant in the descendants from control mothers (Table S3). Interestingly, gene expression of the ChREBP's target lipogenic genes was also induced by tagatose, although in this case, it was significant in the case of males from fructose-fed mothers. With respect to the liver, while Chrebp gene expression was not modified by any treatment, all lipogenic genes regulated by ChREBP (Herman and Birnbaum 2021) that we determined were induced by fructose but not by tagatose intake (Table S3).

### Tagatose induced dyslipidemia in the progeny of fructose-fed dams due to an increased intestinal reabsorption and recirculation of bile acids

**Fig. 3:**
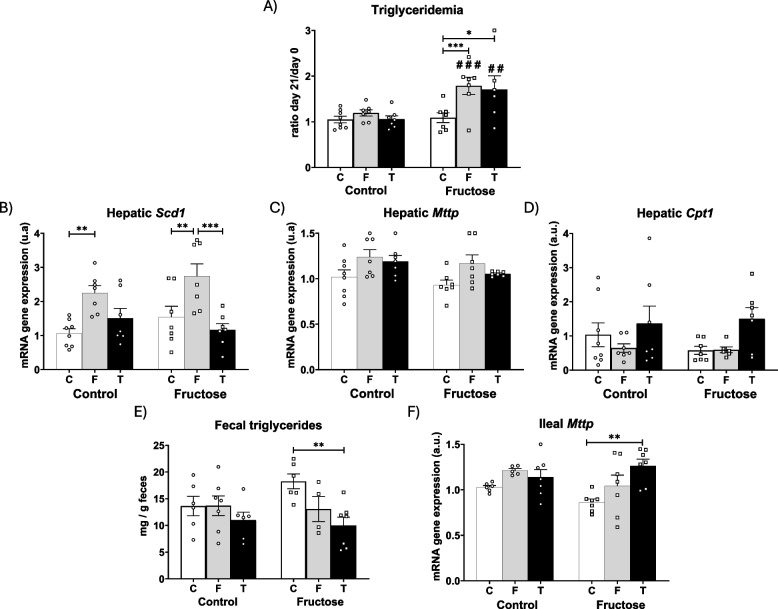
Tagatose produced dyslipidemia in the progeny of fructose-fed dams. **A** ratio day 21/day 0 plasma triglycerides, **B***Scd1*, **C***Mttp*, and **D***Cpt1* mRNA liver gene expression; and **E** fecal triglycerides, and **F***Mttp* mRNA ileal gene expression from control (C, empty bar), fructose- (F, light grey bar), and tagatose-supplemented (T, dark grey bar) male progeny from control (left panel) or fructose-fed (right panel) mothers. Data are means ± S.E. from 7–8 litters. Relative target gene mRNA levels were measured by Real-Time PCR as explained in Materials and Methods, normalized to Rps29 levels and expressed in arbitrary units (a.u.). Asterisks denote a significant difference (*, *p* < 0.05; **, *p* < 0.01; ***, *p* < 0.001) between the groups under the crossbar (groups with a different diet but the same mother´s diet). Hash symbols denote a significant difference (#, *p* < 0.05; ##, *p* < 0.01; ###, *p* < 0.001) as compared to the control mothers (groups with the same diet but different mother´s diet). *Scd1*: stearoyl-CoA desaturase-1; *Mttp*: microsomal triglyceride transfer protein; *Cpt1*: carnitine palmitoyl transferase 1

As described in the previous section, tagatose and fructose were absorbed, reaching the systemic circulation and consequently metabolized by the liver and/or intestine. Since tagatose had been ingested in a significant smaller amount than fructose by all descendants, regardless of maternal diet, it was expected that progeny fed tagatose would not show dyslipidaemia. However, surprisingly, this was not the case. Triglyceridemia was significantly modified after the 21 days of nutritional treatment with both fructose and tagatose and, more importantly, this effect was uniquely observed in progeny from fructose-fed dams (Fig. 3A). It is widely described that fructose is able to raise plasma triglycerides (Tappy and Lê 2010), however, tagatose is considered to improve plasma lipid profile (Smith et al. 2022). These results suggest a worsened lipid profile in descendants of fructose-fed mothers consuming liquid fructose or tagatose. To elucidate the underlying mechanisms of this sugar-induced hypertriglyceridemia, and considering the well-established role of fructose in stimulating lipogenesis and inhibiting β-oxidation, we assessed the hepatic expression of key genes involved in fatty acid metabolism, specifically the lipogenic gene stearoyl-CoA desaturase-1 (*Scd1*) and the catabolic gene carnitine palmitoyl transferase 1 (*Cpt1*) (Tappy and Lê 2010). Thus, fructose intake produced an overexpression of *Scd1* compared to control groups, no matter the mothers' diet, while tagatose consumption did not modify *Scd1* gene expression (Fig. 3B). This increment in fatty acid synthesis caused by fructose could be providing more substrate for triglycerides production and their exportation to blood, leading to an increase in triglyceridemia. Thus, hepatic gene expression of microsomal triglyceride transfer protein (*Mttp*) (Fig. 3C), a protein involved in very low-density lipoprotein (VLDL) assembly, showed a non-significant trend to increase in males consuming fructose, regardless of the diet of their mothers. Related to fatty acid catabolism, *Cpt1* gene expression, the enzyme that controls fatty acid entry to mitochondria for its oxidation, tended to be reduced in males from control mothers that were supplemented with fructose, whereas no changes were observed in the corresponding males from fructose-fed mothers (Fig. 3D). However, tagatose consumption did not provoke any changes in *Cpt1* expression in males from control dams, but it produced a non-significant increase in descendants from fructose-fed mothers (Fig. 3D). Since tagatose intake did not appear to affect lipogenesis, secretion of triglycerides-rich lipoproteins into the bloodstream or fatty acid oxidation (mechanisms that could account for the tagatose-induced hypertriglyceridemia observed in the offspring of fructose-fed mothers), we evaluated alternative pathways.

One of these pathways that could affect the lipid profile in blood is the intestinal absorption of lipids. As shown in Fig. 3E, fecal triglycerides remained unchanged among the three groups of descendants from control mothers. In contrast, in the progeny of fructose-fed dams, sugars intake led to a diminution in fecal triglyceride content, which reached statistical significance in the tagatose group when compared to control animals. Consistent with these findings, ileal gene expression of *Mttp*, the enzyme responsible for assembly in chylomicrons of absorbed lipids, presented no differences in the offspring of control mothers but, importantly, a significant increase was observed in tagatose-supplemented descendants from fructose-fed mothers compared to the control group (Fig. 3F).

**Fig. 4:**
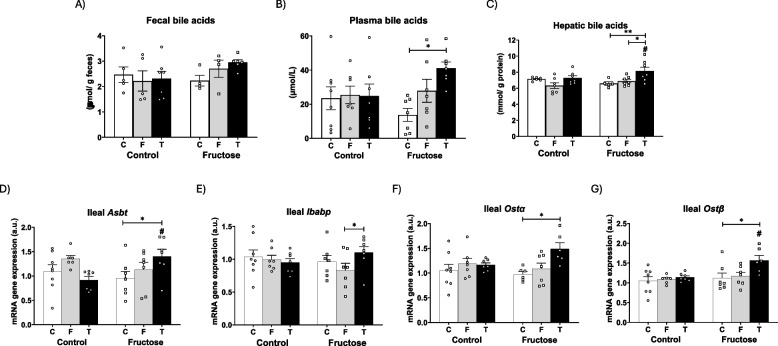
Tagatose produced an increased intestinal reabsorption and recirculation of bile acids in the progeny of fructose-fed dams. **A** Bile acids levels in feces, **B** plasma, **C** and the liver, and **D***Asbt*, **E***Ibabp*, **F***Osta*, and **G***Ostb* mRNA ileal gene expression from control (C, empty bar), fructose- (F, light grey bar), and tagatose-supplemented (T, dark grey bar) male progeny from control (left panel) or fructose-fed (right panel) mothers. Data are means ± S.E. from 7–8 litters. Relative target gene mRNA levels were measured by Real-Time PCR as explained in Materials and Methods, normalized to Rps29 levels and expressed in arbitrary units (a.u.). Asterisks denote a significant difference (*, *p* < 0.05; **, *p* < 0.01; ***, *p* < 0.001) between the groups under the crossbar (groups with a different diet but the same mother´s diet). Hash symbols denote a significant difference (#, *p* < 0.05; ##, *p* < 0.01; ###, *p* < 0.001) as compared to the control mothers (groups with the same diet but different mother´s diet). *Asbt*: apical sodium-bile acid transporter; *Ibabp*: ileal bile acid-binding protein; *Ost*: organic solute transporter

Related to that, bile acids are molecules secreted into bile to facilitate intestinal absorption of fats (Zhou and Hylemon 2014). In the present study, fecal bile acids did not change in descendants of control mothers after consuming fructose or tagatose compared to the control group (Fig. 4A). Interestingly, however, progeny from fructose-fed mothers that received liquid fructose or tagatose did present a non-significant increment in fecal bile acids, being this trend more pronounced in the tagatose group (Fig. 4A). Curiously, this same profile was seen in both plasma (Fig. 4B) bile acids and hepatic (Fig. 4C) bile acids. Thus, in the offspring of fructose-fed mothers that consumed tagatose, plasma bile acids levels were significantly augmented compared to the corresponding control group (Fig. 4B) and hepatic bile acids content (Fig. 4C) was significantly increased in comparison to both control and fructose groups. These results may indicate an alteration in the enterohepatic bile acid recirculation along the intestine-plasma-liver axis. A higher recirculation of bile acids would lead to an increased intestinal content of bile acids which would facilitate lipids emulsification and absorption. This mechanism could contribute to the lipid alterations observed in the offspring of fructose-fed mothers that consumed tagatose. In accordance with this, all genes involved in bile acid reabsorption in the ileum were overexpressed in the progeny from fructose-fed mothers that consumed tagatose (Fig. 4D-G). Thus, whereas in the progeny from control mothers no differences were observed among the three experimental groups, in descendants from fructose-fed mothers, tagatose administration did significantly increase the gene expression of *Asbt* (apical sodium-bile acid transporter) (Fig. 4D) and *Ost* (organic solute transporter), both type alpha (*Osta*) (Fig. 4F) and type beta (*Ostb*) (Fig. 4G) compared to the control group. In the case of *Ibabp* (ileal bile acid-binding protein) gene expression, the significant increase in the tagatose group was found in comparison to males that consumed fructose (Fig. 4E). Moreover, these increases turned out to be also significant for *Asbt* (Fig. 4D) and *Ostb* (Fig. 4G) when comparing males that consumed tagatose from fructose-fed mothers versus descendants of control mothers. Consequently, tagatose intake in descendants from fructose-supplemented mothers would be provoking a higher bile acid entry to the ileum (*Asbt*), an enhanced bile acid circulation across the enterocyte (*Ibabp*), and a more pronounced bile acid export to blood (*Ost*). These results would highlight how foetal programming induced by maternal fructose intake can modulate the response to a nutrient in progeny.

To confirm that the observed dyslipidemia was produced by different mechanisms after consuming fructose than after ingesting tagatose, the gene expression of the transcription factors and their target genes related to the lipogenic pathway was determined in both ileum and liver. As shown in Table S3, in the ileum, whereas *Srebp1c* and *Ppara* were not modified by sugar intake, gene expression of *Srebp1c* target genes showed clear increases in males from fructose-fed mothers that consumed tagatose versus the other two groups. In addition, since cholesterol is necessary to the assembly of lipoproteins and export lipids to the blood, the gene expression of genes related to cholesterol synthesis was measured. A profile similar to that found for lipogenic genes was observed, which would confirm that lipids were being exported from the gut to the blood and contributing to the hyperlipidaemia seen in the offspring of fructose-fed mothers who consumed tagatose. With respect to the liver, the gene expression of *Srebp1c* and *Ppara* was unchanged and, however, the findings observed for the SREBP1c target genes (Table S3) were consistent with those observed in Fig. 3B and C.

### Tagatose modified plasma FGF21 but not GLP1 levels in the progeny independently of the mother’s diet

Intestinal production of triglyceride-rich lipoproteins has also been shown to be affected by glucagon-like peptide 1 (GLP-1) (Mulvihill 2018), an incretin produced by the gut that has been reported to play a key role in inducing satiety, reducing food intake and controlling obesity. That is why the study of this molecule is attracting increasing interest among the healthcare scientific community. Interestingly, tagatose has been shown to stimulate the release of GLP-1 (Smith et al. 2022), although short-chain fatty acids (SCFA) produced by bacterial fermentation of poorly absorbed sugars into the gut may also stimulate GLP-1 (Guerrero-Wyss et al. 2018). In fact, GLP-1 is released in response to the presence of diverse nutrients in the intestine. Expression level of the ileal proglucagon gene (a precursor of GLP-1) has been shown to be increased in the presence of glucose, free fatty acids and SCFA in the distal gut (Dantas Machado et al. 2022).

**Fig. 5:**
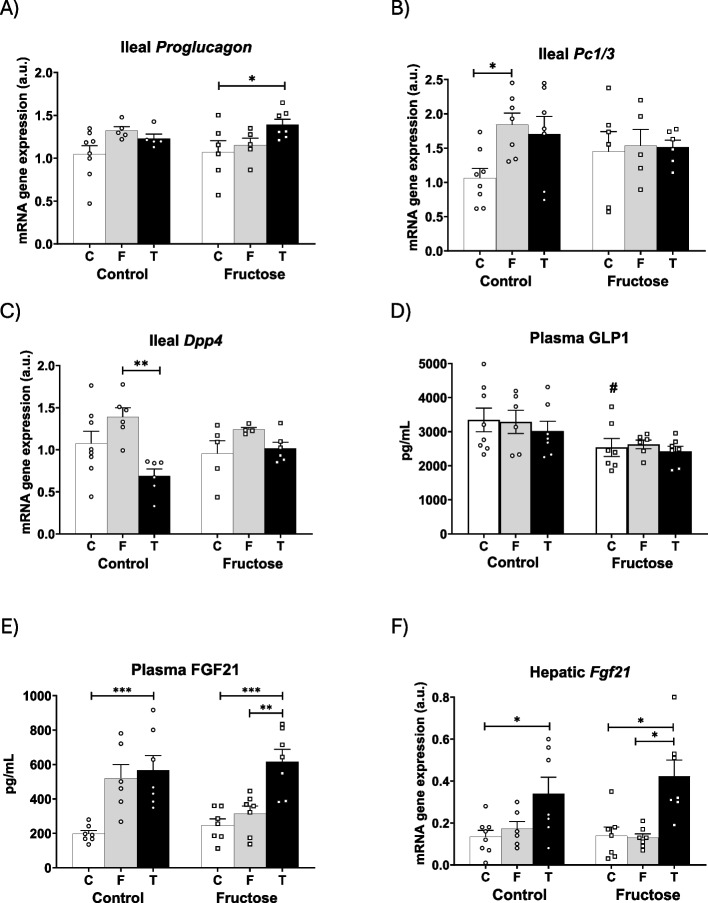
Tagatose modified plasma FGF21 but not GLP1 levels in the progeny independently of the mother´s diet. **A** Proglucagon, **B***Pc1/3* and **C***Dpp4* mRNA ileal gene expression, and **D** plasma GLP1 and **E** FGF21 levels, and **G***Fgf21* mRNA hepatic gene expression from control (C, empty bar), fructose- (F, light grey bar), and tagatose-supplemented (T, dark grey bar) male progeny from control (left panel) or fructose-fed (right panel) mothers. Data are means ± S.E. from 7–8 litters. Relative target gene mRNA levels were measured by Real-Time PCR as explained in Materials and Methods, normalized to Rps29 levels and expressed in arbitrary units (a.u.). Asterisks denote a significant difference (*, *p* < 0.05; **, *p* < 0.01; ***, *p* < 0.001) between the groups under the crossbar (groups with a different diet but the same mother´s diet). Hash symbols denote a significant difference (#, *p* < 0.05; ##, *p* < 0.01; ###, *p* < 0.001) as compared to the control mothers (groups with the same diet but different mother´s diet). *Pc1/3*: proprotein convertase 1/3; *Dpp4*: dipeptidyl peptidase 4; GLP1: glucagon-like protein 1; FGF21: fibroblast growth factor 21

In the present study, tagatose intake led to an increased ileal gene expression of transporters/receptors of nutrients such as *Glut5* (Fig. 2A) for sugars, *Abst* (Fig. 4D) for bile acids, G protein-coupled receptors (*Gpr41* and *Gpr43*) for SCFAs, and Takeda G protein-coupled receptor (*Tgr5*) for bile acids (Table 1), being the effect more evident in the progeny of fructose-fed mothers. Interestingly, the upregulation of these genes may help to explain why proglucagon gene expression was induced in descendants of fructose-fed dams consuming tagatose (Fig. 5A), reaching statistical significance in comparison to the control group. However, this increase in proglucagon gene expression in males from fructose-fed mothers consuming tagatose was not accompanied by an augmented expression of *Pc1/3* (proprotein convertase 1/3) (Fig. 5B), the enzyme responsible for processing proglucagon to the active form of GLP1, nor by a decreased expression of *Dpp4* (dipeptidyl peptidase 4), the enzyme that degrades GLP-1 (Fig. 5C). Consequently, plasma GLP1 levels did not differ among all experimental groups (Fig. 5D). It is important to note that we could not measure active GLP-1 levels in serum and the data shown in Fig. 5D reflect total GLP-1 levels.

**Table 1:** Ileal and hepatic gene (mRNA) expression of control (C), fructose- (F), and tagatose-supplemented (T) male progeny from control or fructose-fed mothers

	CONTROL MOTHERS				FRUCTOSE MOTHERS			
	CONTROL	FRUCTOSE	TAGATOSE	*p*	CONTROL	FRUCTOSE	TAGATOSE	*p*
Ileal mRNA Gene Expression (a.u)								
*Gpr41*	1.033 ± 0.104	1.213 ± 0.128	1.264 ± 0.116		0.761 ± 0.076	0.718 ± 0.111#	1.255 ± 0.192	* (FC vs FT) * (FF vs FT)
*Gpr43*	1.057 ± 0.129	1.468 ± 0.1778	1.637 ± 0.205	* (CC vs CT)	1.069 ± 0.131	1.039 ± 0.203	1.448 ± 0.132	
*Tgr5*	1.132 ± 0.087	1.78 ± 0.178	1.687 ± 0.263	* (CC vs CF)	1.082 ± 0.063	1.313 ± 0.138	1.548 ± 0.076	** (FC vs FT)
Hepatic mRNA Gene Expression (a.u)								
*Mct1*	1.002 ± 0.077	1.048 ± 0.104	0.977 ± 0.043		1.158 ± 0.112	1.044 ± 0.056	1.335 ± 0.127##	
*Hdac1*	1.024 ± 0.08	1.003 ± 0.136	1.290 ± 0.105		0.975 ± 0.047	0.868 ± 0.121	1.062 ± 0.095	
*Hdac3*	0.943 ± 0.057	0.990 ± 0.092	1.034 ± 0.096		1.055 ± 0.086	1.042 ± 0.048	0.882 ± 0.04	
*Pdk4*	1.080 ± 0.162	1.003 ± 0.010	1.583 ± 0.196		1.067 ± 0.109	0.993 ± 0.178	1.483 ± 0.086	
*Cidec*	1,008 ± 0,048	1.105 ± 0.108	1.360 ± 0.089	* (CC vs CT)	1.106 ± 0.076	1.100 ± 0.123	1.374 ± 0.087	
*Vldlr*	1.037 ± 0.096	0.883 ± 0.042	1.033 ± 0.113		1.679 ± 0.257	2.035 ± 0.388##	1.945 ± 0.271##	
*Cd36*	0.0014 ± 0.0002	0.0016 ± 0.0001	0.0024 ± 0.0002		0.0022 ± 0.0006	0.0017 ± 0.0004	0.0011 ± 0.0002#	

Ileal and hepatic levels of specific mRNA genes are shown. Ileum mRNA expression represents GLP1 signalling pathway genes, and liver mRNA expression represents both SCFA and PPAR alpha signalling pathway genes. Relative target gene mRNA levels were measured by Real Time PCR as explained in Materials and Methods, normalized to Rps29 levels and expressed in arbitrary units (a.u.). Data are means ± S.E. from 7 to 8 litters. Asterisks denote a significant difference (*, *P* < 0.05; **, *P* < 0.01) between the groups with a different diet but the same mothers ´ diet. Hash symbols denote a significant difference (#, *P* < 0.05; ##, *P* < 0.01) as compared to the control mothers (groups with the same diet but different mothers ´ diet). fructose. The first letter indicates whether the mothers had been supplied with tap water during pregnancy (C: control) or liquid fructose (F); and the second letter indicates the nutritional treatment without (C: control) or with additives, fructose (F) or tagatose (T), when they were adults

*GLP1* Glucagon-like protein 1, and *GLP1* signalling, *Gpr* G protein-coupled receptors, *Tgr* Takeda G protein-coupled receptor, *SCFA* Short-chain fatty acids, and *SCFA* signalling, *Mct1* Monocarboxylate transporter 1 protein, *Hdac* Histone deacetylases, *PPAR* Peroxisome proliferator-activated receptor, and *PPAR* signalling, *Pdk* Pyruvate dehydrogenase kinase, *Cidec* Cell death-inducing DNA fragmentation factor-like effector C, *Vldlr* Very low-density lipoprotein receptor, *Cd36* Cluster of differentiation 36

On the other hand, it has been reported that FGF21, a hormone that also responds to nutrients, can regulate sugar preference and, in addition, its production is induced by GLP-1 analogues (Kim and Lee 2015). In the present work we found that FGF21 plasma levels were augmented in sugar-supplemented descendants, regardless of the diet consumed by their mothers (Fig. 5E). Curiously, plasma FGF21 levels paralleled the different amount of liquid fructose ingested by descendants, which was influenced by their mothers´diet (Fig. 1A). Importantly, tagatose-fed descendants, who had consumed approximately 10 times less amount of sugar than the fructose-fed groups, showed elevated plasma FGF21 levels. This increase in the tagatose-supplemented animals was significant compared to the control group in the progeny of control mothers and compared to the control and fructose-fed groups in descendants from fructose-fed dams (Fig. 5E). Considering that FGF21 is mainly synthesized in the liver and that our results indicate that tagatose, unlike fructose, barely reaches this organ, it was very striking to find that tagatose intake induced hepatic *Fgf21* gene expression whereas fructose did not. Moreover, this tagatose-induced activation was more pronounced in the offspring of fructose-fed dams than in those of control mothers (Fig. 5F).

### Tagatose intake increased plasma levels of Ang II which produced worse effects in the offspring of fructose-fed mothers

It has been shown that, in response to carbohydrate intake, the liver produces FGF21 which acts on the hypothalamus to selectively suppress sugar intake. Interestingly, in the present study, after tagatose consumption, plasma FGF21 levels and sugar intake were inversely related, that is, tagatose increased plasma FGF21, and this reduced sugar appetite (Fig. 5E versus Fig. 1A). In contrast, in fructose-fed animals we found a direct relationship between sugar intake and FGF21 levels. Carbohydrates are known to activate hepatic ChREBP, a transcription factor that promotes FGF21 production in the liver (Holstein-Rathlou et al. 2016). However, as previously mentioned, tagatose consumption hardly affected the hepatic expression of some ChREBP target genes such as *Khk* and *AldoB* (Fig. 2F and G). These data suggest that ChREBP is likely not involved in the tagatose-induced activation of hepatic FGF21 production.

Tagatose is minimally absorbed in the intestine and, thus, it has been proposed as a prebiotic agent capable of selectively stimulating the growth of specific gut microbiota and affecting host health (Son et al. 2019). By doing this, tagatose induces microbiota that produce beneficial compounds such as butyrate. This SCFA has been demonstrated to stimulate hepatic FGF21 gene expression by inhibiting histone deacetylase 3 (*Hdac3*) which suppresses the activity of peroxisome proliferator-activated receptor type alpha (PPARα) (Li et al. 2012). And, to note, FGF21 is a well-known PPAR alpha target gene in liver (Rakhshandehroo et al. 2010). However, none of the nutritional interventions here used altered the gene expression of monocarboxylate transporter 1 (*Mct1*), which permits SCFA entry to the cell, or that of *Hdac1* or *Hdac3* (Table 1). Thus, exploring the role of PPARα in the tagatose-induced activation of FGF21, we found that whereas *Cpt1* (Fig. 3D), *Pdk4* and *Cidec* gene expression (Table 1) observed in the tagatose groups could reflect an activation of PPARα, the findings observed for *Scd1* (Fig. 3B), *Vldlr* and *Cd36* gene expression did not show changes in that sense (Table 1). Moreover, it has been suggested that part of the effect of PPARα on hepatic ketogenesis may be mediated by induction of the PPARα target FGF21; however, plasma ketone bodies trended to be diminished in all descendants consuming carbohydrates regardless of their mothers´diet [166.1 ± 28.4; 92.9 ± 19.3; and 95.6 ± 5.1 for control, fructose- and tagatose-fed descendants from control mothers; 206.3 ± 41.2; 143.1 ± 41.1; and 165.7 ± 52.3 mM for control, fructose- and tagatose-fed males from fructose-fed dams].

**Fig. 6:**
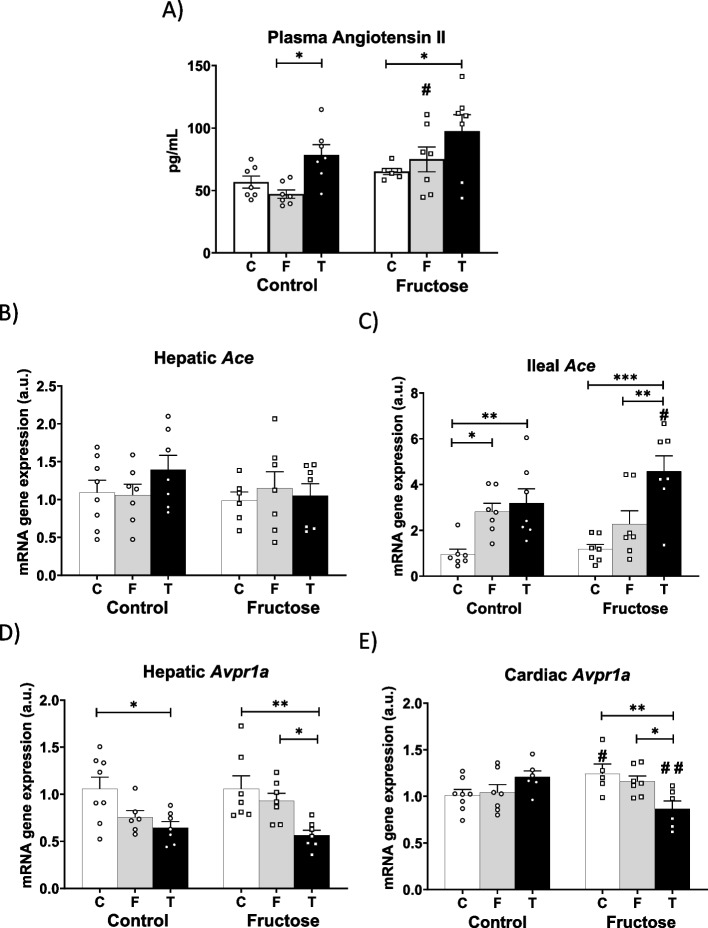
Tagatose modified plasma Ang II levels in the progeny regardless of the diet of the mother. **A** Plasma angiotensin II; **B** hepatic *Ace* and **C** ileal *Ace* mRNA gene expression; and **D** hepatic *Avpr1a* and **E** cardiac *Avpr1a* mRNA gene expression from control (C, empty bar), fructose- (F, light grey bar), and tagatose-supplemented (T, dark grey bar) male progeny from control (left panel) or fructose-fed (right panel) mothers. Data are means ± S.E. from 7–8 litters. Relative target gene mRNA levels were measured by Real-Time PCR as explained in Materials and Methods, normalized to Rps29 levels and expressed in arbitrary units (a.u.). Asterisks denote a significant difference (*, *p* < 0.05; **, *p* < 0.01; ***, *p* < 0.001) between the groups under the crossbar (groups with a different diet but the same mother´s diet). Hash symbols denote a significant difference (#, *p* < 0.05; ##, *p* < 0.01; ###, *p* < 0.001) as compared to the control mothers (groups with the same diet but different mother´s diet). Ang II: angiotensin II; *Ace*: angiotensin I converting enzyme; *Avpr1a*: arginine vasopressin receptor 1 A gene

Importantly, angiotensin II (Ang II), a molecule that promotes inflammation, oxidative stress, vascular injury, fatty liver and insulin resistance (Mastoor et al. 2022), has been shown to be able to increase serum FGF21 levels and hepatic *Fgf21* gene expression, possibly as a compensatory and protective response against these harmful effects induced by Ang II (Pan et al. 2018; Li et al. 2019). As shown in Fig. 6A, and in accordance with the results found in hepatic *Fgf21* gene expression (Fig. 5G), tagatose intake produced an increase in plasma Ang II in both descendants from control mothers and males from fructose-fed dams. This augmentation observed in tagatose-fed progeny turned out to be significant versus fructose-fed animals in descendants of control mothers and versus the control group in males from fructose-fed mothers. In accordance, the angiotensin-converting enzyme (ACE), that converts AngI to AngII, although its hepatic gene expression was not affected by any dietary treatment (Fig. 6B), ileal *Ace* gene expression was significantly augmented after carbohydrate ingestion in males from control mothers and sharply increased after tagatose intake in progeny from fructose-fed dams. In fact, these tagatose-mediated effects were significant when compared to the other two groups of descendants from fructose-fed dams. Moreover, tagatose-supplemented males from fructose-fed mothers showed a significant higher expression of ileal *Ace* than the descendants of control mothers fed with the same sugar (denoted by a hash symbol) suggesting a fetal programming mediated effect (Fig. 6C).

Additionally, Ang II is well-known to stimulate vasopressin secretion (Reid et al. 1983), a molecule that is related to liquid ingestion, carbohydrate (fructose, glucose or HFCS) intake and it has been proposed as a key mediator in the fructose-induced metabolic syndrome (Andres-Hernando et al. 2021). However, we measured plasma copeptin, an analogue and stable peptide derived from the precursor of vasopressin, and no differences were found among all the experimental groups [46.1 ± 6.8; 36.8 ± 5.4; and 49.9 ± 4.9 for control, fructose- and tagatose-fed descendants from control mothers; 49.4 ± 6.9; 46.0 ± 0.8; and 39.9 ± 3.0 pg/mL for control, fructose- and tagatose-fed males from fructose-fed dams]. Nevertheless, despite this lack of effect mediated by carbohydrate intake in copeptin levels, the mRNA gene expression of vasopressin 1a receptor (*Avpr1a*) showed a reduction in the liver of fructose-fed animals from control mothers in consonance to previous studies (Andres-Hernando et al. 2021) and a significant reduction in tagatose-supplemented males from these same mothers (Fig. 6D). Curiously, in the progeny from fructose-fed mothers, the effect produced by fructose ingestion was not observed whereas tagatose intake significantly diminished *Avpr1a* expression versus both control and fructose groups. Furthermore, this fetal programming effect was more evident in heart *Avpr1a* expression (Fig. 6E), since no changes were observed among the three experimental groups of descendants from control mothers, but a significant reduction was found in tagatose-fed males from fructose-fed mothers in comparison to the other two groups and also when compared to the corresponding tagatose-fed group from control mothers (hash symbol). Interestingly, Andres-Hernando et al. observed in AVP1AR-KO mice worse metabolic features of metabolic syndrome than in the wild-type group (Andres-Hernando et al. 2021).

**Fig. 7:**
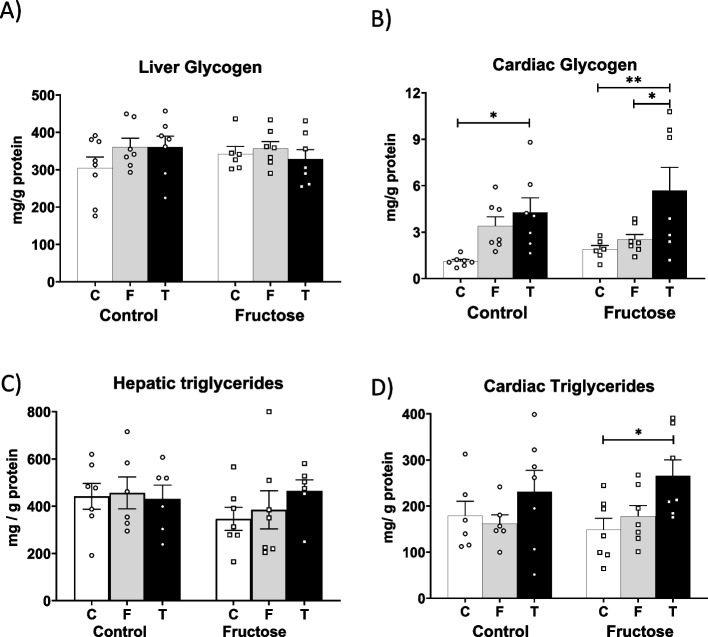
Tagatose produced cardiac accretion of glycogen and triglycerides mainly in fructose-fed mother descendants. **A** hepatic and **B** cardiac glycogen contents, and **C** hepatic and **D** cardiac triglycerides from control (C, empty bar), fructose- (F, light grey bar), and tagatose-supplemented (T, dark grey bar) male progeny from control (left panel) or fructose-fed (right panel) mothers. Data are means ± S.E. from 7–8 litters. Relative target gene mRNA levels were measured by Real-Time PCR as explained in Materials and Methods, normalized to Rps29 levels and expressed in arbitrary units (a.u.). Asterisks denote a significant difference (*, *p* < 0.05; **, *p* < 0.01; ***, *p* < 0.001) between the groups under the crossbar (groups with a different diet but the same mother´s diet). Hash symbols denote a significant difference (#, *p* < 0.05; ##, *p* < 0.01; ###, *p* < 0.001) as compared to the control mothers (groups with the same diet but different mother´s diet)

In view of these interesting results observed in tagatose-fed males, that is, elevated levels of plasma FGF21 trying to compensate the high levels of plasma Ang II and a lower presence of the vasopressin 1a receptor, we decided to evaluate oxidative stress and lipid and glucose dysfunction parameters in both liver and heart. Curiously, FGF21 appeared to counteract the effects of Ang II in the liver but not in the heart. This protective effect was more evident in the progeny from control mothers than in those from fructose-fed dams. Thus, in the liver, none of the nutritional interventions altered MDA levels, catalase activity or superoxide dismutase activity, regardless of the maternal diet (Table 2). In contrast, in the heart, there was a tendency for these oxidative markers to be modified by carbohydrate intake in progeny from control mothers, although statistically significant differences were only found in tagatose-fed descendants from fructose-fed mothers. Specifically, catalase activity was significantly decreased compared to the fructose group, while SOD activity was elevated versus both fructose and control groups (Table 2). As shown in Fig. 7A, whereas liver glycogen content remained unchanged in all experimental groups, cardiac glycogen content (Fig. 7B) was augmented by carbohydrate intake in the progeny from control mothers, becoming significantly different in tagatose-fed males versus the control group. This tagatose-mediated increase in cardiac glycogen was even more pronounced and significant in descendants from fructose-fed dams versus the other two groups. Additionally, a similar profile to the one observed in oxidative parameters and glycogen was also found in the triglycerides content. Thus, hepatic steatosis was not observed in any case, although a slight non-significant lipid accretion was found in tagatose-fed animals from fructose-fed dams (Fig. 7C). Interestingly, whereas cardiac triglycerides content did not change among the three experimental groups in males from control dams, a significant triglycerides deposit was found in males from fructose-fed dams after consuming tagatose (Fig. 7D) compared to the control group.

**Table 2:** Hepatic and cardiac oxidative stress parameters of control (C), fructose- (F), and tagatose-supplemented (T) male progeny from control or fructose-fed mothers

	CONTROL MOTHERS				FRUCTOSE MOTHERS			
	CONTROL	FRUCTOSE	TAGATOSE	*p*	CONTROL	FRUCTOSE	TAGATOSE	*p*
Liver oxidative-stress parameters								
MDA (mmol/g tissue)	15.552 ± 1.357	15.398 ± 1.737	17.383 ± 1.616		14.553 ± 0.463	14.447 ± 0.664	18.227 ± 1.924	
Cat (mU/mg prot)	1391.6 ± 120.8	1036.4 ± 103.7	1373.4 ± 117.8		1578.1 ± 121.5	1682.3 ± 190.1##	1663.9 ± 232.6	
SOD (U/mg prot)	43.33 ± 2.67	42.37 ± 1.43	44.35 ± 1.67		40.79 ± 0.47	43.11 ± 0.33	44.65 ± 2.32	
Heart oxidative-stress parameters								
MDA (nmol/g tissue)	36.17 ± 4.21	52.33 ± 7.34	57.87 ± 12.77		34.38 ± 4.53	48.70 ± 12.83	38.55 ± 5.64	
Cat (mU/mg prot)	42.91 ± 2.65	39.52 ± 2.42	36.07 ± 2.20		40.67 ± 1.08	43.48 ± 1.90	33.82 ± 1.54	** (FF vs FT)
SOD (U/mg prot)	23.54 ± 0.52	24.57 ± 0.49	27.04 ± 1.38		23.78 ± 0.92	23.42 ± 1.51	28.83 ± 1.21	* (FC vs FT) ** (FF vs FT)

Data are means ± S.E. from 7 to 8 litters. Asterisks denote a significant difference (*, *p* < 0.05; **, *p* < 0.01) between the groups with a different diet but the same mothers ´ diet. Hash symbols denote a significant difference (##, *p* < 0.01) as compared to the control mothers (groups with the same diet but different mothers ´ diet). fructose. The first letter indicates whether the mothers had been supplied with tap water during pregnancy (C: control) or liquid fructose (F); and the second letter indicates the nutritional treatment without (C: control) or with additives, fructose (F) or tagatose (T), when they were adults

*MDA* Malondialdehyde, *Cat* Catalase activity, *SOD* Superoxide dismutase activity

Therefore, this oxidative stress and accumulation of fuel stores suggest that tagatose consumption induces cardiac metabolic dysregulation in the offspring of fructose-fed mothers (Varma et al. 2018; Ye et al. 2004) that ultimately would lead to cardiac dysfunction. Moreover, we have previously described a similar scenario in which FGF21 was able to protect against lipid accretion and oxidative stress influenced by maternal nutrition and in a tissue-dependent manner (Fauste et al. 2020).

## Discussion

The urgent need to reduce the high prevalence of metabolic diseases, especially those driven by modifiable lifestyle factors such as "diseases related to processed food", makes dietary changes essential. One key aspect of these changes involves reducing added sugars intake, particularly fructose or its derivatives, and replacing them with alternative sugars or sweeteners, such as rare sugars. This shift has already been taking place since the first decades of this century and, fortunately, it has contributed to a gradual slowdown in the development of metabolic diseases. However, the decrease in added sugar consumption has not been paralleled by a proportional reduction in the incidence of metabolic diseases such as obesity (Faruque et al. 2019). We believe this discrepancy is because high fructose consumption was common among pregnant women prior to these dietary changes. Consequently, it is logical that the offspring of these mothers continue to exhibit a high incidence of metabolic diseases and that their response to diet is affected by a fetal programming mechanism due to the maternal intake of added sugar (Gracner et al. 2024). Given the increasing incorporation of rare sugars to replace fructose, sucrose or HFCS in our diet, we decided to find out the impact of maternal fructose intake on offspring´s metabolic response to consumption of the rare sugar tagatose.

One of the most striking results found is that, despite the low intake of tagatose (about 5% of total caloric intake), it was able to stimulate the gene expression of enzymes and transporters of the tagatolysis pathway in the intestine, in a more pronounced way in descendants of fructose-fed mothers, and, interestingly, with almost no influence on hepatic tagatolysis. In contrast, fructose, which accounted for about 45% of total caloric intake, had the opposite effect, that is, it did not modify intestinal fructolysis and clearly affected the gene expression of liver fructolysis enzymes and transporters (Andres-Hernando et al. 2020). These data suggest that the effects observed are more related to a metabolite produced by intestinal tagatolysis than to tagatose itself.

Another relevant finding was that tagatose intake affected triglyceridemia in a way that was clearly influenced by fetal programming, since it induced hypertriglyceridemia in the offspring of fructose-fed mothers, but not in those of control mothers. Fructose intake also elevated triglyceridemia in that same group of descendants. Interestingly, while fructose-mediated effect was mainly caused by a higher expression of lipogenic genes in the liver, the effect of tagatose appeared to be associated with a higher gene expression of bile acid transporters in the intestine. This result suggests a greater enterohepatic recirculation of bile acids, which led to a greater lipid absorption and packaging of in the intestine. Such a mechanism would explain the hyperlipidemia observed in these descendants and aligns with findings previously described by us and others although using another type of diet (Fauste et al. 2024; Downing et al. 2017).

Interestingly, gene expression of most transporters and receptors of nutrients, such as sugars, short-chain fatty acids, and bile acids, was increased in the ileum after tagatose intake, particularly in the offspring of fructose-fed dams. These transporters and receptors have been directly related to intestinal production of proglucagon in response to these nutrients (Dantas Machado et al. 2022). In fact, consistent with this, we observed that the tagatose-induced upregulation of the expression of nutrient transporters and receptors was accompanied by an increase in the expression of proglucagon. However, plasma GLP1 levels did not reflect this observed rise in its precursor molecule. In addition, GLP2, which is also produced from proglucagon and whose high levels would be more in line with the greater lipid absorption and packaging found in the descendants of fructose-fed mothers consuming tagatose, was not affected by the different treatments (Mulvihill 2018). Unfortunately, total GLP was measured and not the active form that would be more informative.

One of the most striking results was to find that both tagatose intake and, as expected, fructose consumption increased plasma levels of FGF21 (Holstein-Rathlou et al. 2016). Interestingly, although tagatose (as mentioned above) seemed to barely reach the liver as it failed to stimulate tagatolysis in this organ, it was able to clearly increase the hepatic expression of *Fgf21*. FGF21 has been described as a molecule capable of decreasing the sweet taste preference (Holstein-Rathlou et al. 2016). Therefore, it was paradoxical to find that, whereas fructose intake was directly related to plasma FGF21 levels, the relationship with tagatose consumption was clearly opposite and more in line with what has been previously described, that is, the tagatose-induced increase in FGF21 could be the cause of the low intake of this sugar observed in the offspring.

We were therefore interested in discovering the mechanism by which tagatose intake increases the hepatic expression of FGF21. We found that classic effectors such as ChREBP, PPAR alpha and even the mediation of SCFA that could be produced in the intestine by unabsorbed tagatose, failed to explain it adequately (Lu et al. 2008). However, a less studied regulatory molecule in the metabolism of FGF21, such as angiotensin II, was found to be increased in plasma after tagatose intake. This increase was consistent with the findings observed in hepatic expression of *Fgf21*, so we could affirm that tagatose consumption increased Ang II levels and, with it, hepatic production of FGF21.

Interestingly, Ang II has also been shown to be involved in the production of vasopressin (Reid et al. 1983), a molecule that could influence the lower fluid intake seen in animals that consumed tagatose, as has been described in the case of fructose (Andres-Hernando et al. 2021). However, levels of copeptin (a stable form of vasopressin) were not changed by any of the treatments. On the other hand, the hepatic expression of its receptor 1a (*Avpr1a*) was modified by fructose intake, which decreased it, confirming previous findings by other authors (Andres-Hernando et al. 2021). Notably, tagatose reduced the hepatic gene expression of this receptor more drastically than fructose in all descendants and, furthermore, it also diminished Avpr1a gene expression in the heart, although in this case this effect was only observed in descendants of fructose-mothers. Considering that the lack of this receptor has been related to the appearance of more severe symptoms of metabolic syndrome, this interesting result warrants further investigation in future studies (Andres-Hernando et al. 2021).

**Fig. 8:**
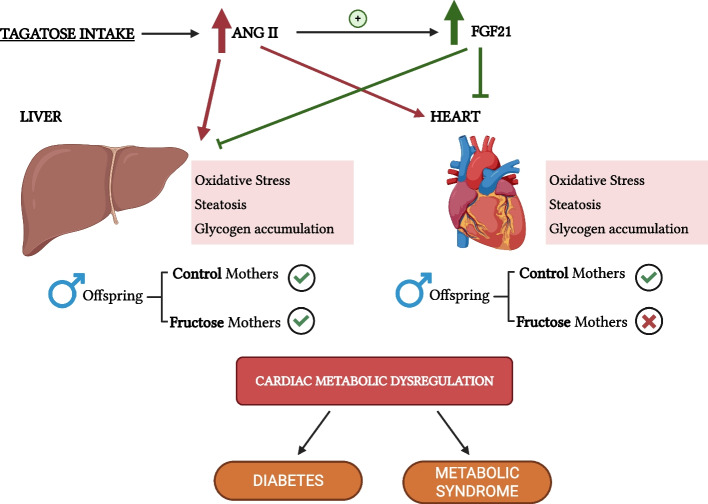
Diagram explaining maternal diet intake-dependent derangements observed after tagatose consumption in descendants and their association with development of Metabolic Syndrome and/or diabetes. Oxidative stress and accumulation of glycogen and triglycerides were observed in heart after the intake of tagatose in males from fructose-fed mothers. These dysfunctions have been related in other studies with cardiac metabolic dysregulation, a very common situation observed in diabetes or Metabolic Syndrome. Green ticks indicate when FGF21 was able to counteract the negative effects of Ang II; red cross indicates when FGF21 could not counteract the harmful effects of Ang II. Created with BioRender.com

It has been described that Ang II promotes the production of FGF21 to counteract its adverse effects (Pan et al. 2018; Li et al. 2019). Thus, in the present study, FGF21 manages to counteract the negative effects of Ang II in the liver of all descendants fed tagatose. However, this was not the case in the heart of males from fructose-fed mothers since oxidative stress and a clear accumulation of glycogen and triglycerides were observed after the intake of tagatose. These dysfunctions could be an initial biomarker of cardiac metabolic dysregulation, a very common situation observed in diabetes or metabolic syndrome (Varma et al. 2018). Figure 8 shows a diagram explaining these results in detail.

Overloaded fuel storage is an evident feature in the diabetic heart, with accumulation of glycogen and lipid droplets. Cardiac metabolic dysregulation in diabetes has been linked to oxidative stress and impaired autophagy, which can lead, among other disturbances, to cardiac dysfunction (Varma et al. 2018). All of these mechanisms have been found to link cardiac dysfunction and metabolic syndrome(Ilkun and Boudina 2013). Our findings are therefore quite interesting because they contrast with several recent studies on tagatose intake. Thus, Durante et al. (2021), using 30% tagatose chronically in rats, found that this sugar could be considered an alternative to saccharose, as tagatose produced a better metabolic profile and lower cardiac susceptibility to ischemia–reperfusion injury compared to its isomer fructose(Durante et al. 2021). In addition, Sambra et al. (2022) observed that tagatose may have beneficial metabolic effects in women with insulin resistance, while stevia promoted harmful insulinotropic consequences in the same experimental group (Sambra et al. 2022). Therefore, these controversies over the effects of tagatose consumption indicate the need for further studies to clarify this and, possibly, a reconsideration of this rare sugar as GRAS.

Limitations of this study, apart from difficulties in extrapolating results from experimental animals to humans, include: We have used simple sugars solutions, and, in our society, simple sugars are rarely taken separately. We have not explored any other causes of low tagatose intake other than FGF21, i.e., intestinal glucose production and its postgastric signaling, tagatose-1P accumulation and sugar aversion, an intestine-brain-liver nerve pathway or an impaired intestinal sugar absorption and its gastrointestinal side effects. We have not investigated whether the effects observed in males consuming tagatose are due to tagatose itself or to some metabolite produced from tagatose in the intestine, such as glycerate. And finally, we have studied ileal metabolism, but it has been shown that fructose is preferentially metabolized in the jejunum, whereas tagatose, possibly, in the ileum. Nevertheless, it has been reported that fructose is also metabolized even in the cecum (Jang et al. 2020).

## Conclusions

Therefore, the consumption of alternative sugars to fructose and/or HFCS, such as tagatose, by descendants of mothers who consumed fructose during their pregnancy could lead to adverse effects more likely than in descendants of control mothers.

The current study highlights a novel interaction between tagatose consumption and angiotensin II signaling, leading to increased hepatic FGF21 expression even in the absence of direct hepatic sugar metabolism. This unique metabolic response raises concerns regarding its systemic effects, particularly in individuals with prenatal exposure to excess fructose. The inability of FGF21 to mitigate Ang II-associated dysfunction in cardiac tissue suggests early biomarkers of metabolic disruption. These data call for a re-evaluation of the metabolic safety of rare sugars like tagatose.

Overall, these findings emphasize the key role of maternal nutrition during gestation, particularly the quality and quantity of sugar intake, as it can seriously affect the long-term metabolic health of the progeny through fetal programming mechanisms. Consequently, these observations along with previous reports strongly support the adherence to the WHO guidelines which recommend limiting the intake of simple sugars in processed foods and sugary drinks to less than 10% of total daily caloric intake. Importantly, as evidenced by the present study, such limitations should also include rare sugars such as tagatose.

## Supplementary Information

Supplementary Material 1.

Supplementary Material 2.

Supplementary Material 3.

## Data Availability

All data generated or analysed during this study are included in this published article [and its supplementary information files].
