# Supplementary material for: Tagatose consumption provokes metabolic syndrome features in rat males from mothers that consumed fructose during their pregnancy
Source: Mol Med. 2025 Dec 29;31:339. doi: 10.1186/s10020-025-01402-3 (PMC12751481; doi:10.1186/s10020-025-01402-3)
Supplement: Supplementary file 1 — Supplementary Material 1. [file 10020_2025_1402_MOESM1_ESM.docx]

| **2-way ANOVA** | AUC ingested liquid (mL/21 days per rat) | | AUC consumed diet (g/21 days per rat) | | Total amount of ingested energy (KCal/21 days per rat) | | Body weight increase (g) | | Body weight at day 21 (g) | | Body weight  at day 0 (g) | | |
| --- | --- | --- | --- | --- | --- | --- | --- | --- | --- | --- | --- | --- | --- |
|  | p | η2 | p | η2 | p | η2 | p | η2 | p | η2 | | p | η2 |
| **M** | 0,109 | 0,144 | 0,478 | 0,030 | 0,658 | 0,011 | 0,706 | 0,004 | 0,000 | 0,393 | | 0,000 | 0,519 |
| **D** | 0,000 | 0,905 | 0,000 | 0,622 | 0,000 | 0,638 | 0,001 | 0,331 | 0,685 | 0,020 | | 0,080 | 0,128 |
| **MxD** | 0,041 | 0,313 | 0,313 | 0,128 | 0,332 | 0,115 | 0,297 | 0,065 | 0,199 | 0,084 | | 0,218 | 0,079 |

| **2-way ANOVA** | Liver weight (g) /Body weight(g) | | Heart weight (g) /Body weight(g) | | WAT (g)/Body weight(g) | | Ileal GLUT5  (au) | | Ileal KHK  (au) | | | Ileal AldoB  (au) | | |
| --- | --- | --- | --- | --- | --- | --- | --- | --- | --- | --- | --- | --- | --- | --- |
|  | p | η2 | p | η2 | p | η2 | p | η2 | p | | η2 | p | η2 | |
| **M** | 0,028 | 0,124 | 0,036 | 0,123 | 0,004 | 0,205 | 0,059 | 0,104 | 0,075 | 0,083 | | 0,370 | | 0,022 |
| **D** | 0,031 | 0,171 | 0,903 | 0,006 | 0,326 | 0,060 | 0,000 | 0,641 | 0,004 | 0,262 | | 0,000 | | 0,369 |
| **MxD** | 0,518 | 0,035 | 0,290 | 0,070 | 0,078 | 0,132 | 0,466 | 0,045 | 0,766 | 0,014 | | 0,260 | | 0,072 |

| **2-way ANOVA** | Ileal TFKC (au) | | Liver GLUT5 (au) | | Liver KHK (au) | | Liver AldoB (au) | | Liver TFKC (au) | | Triglyceridemia  (mg/dL) | | Liver SCD1 (au) | |
| --- | --- | --- | --- | --- | --- | --- | --- | --- | --- | --- | --- | --- | --- | --- |
|  | p | η2 | p | η2 | p | η2 | p | η2 | p | η2 | p | η2 | p | η2 |
| **M** | 0,651 | 0,006 | 0,007 | 0,188 | 0,850 | 0,001 | 0,950 | 0,000 | 0,536 | 0,011 | 0,054 | 0,097 | 0,332 | 0,025 |
| **D** | 0,000 | 0,375 | 0,000 | 0,587 | 0,000 | 0,472 | 0,003 | 0,287 | 0,000 | 0,495 | 0,094 | 0,120 | 0,000 | 0,422 |
| **MxD** | 0,253 | 0,076 | 0,441 | 0,046 | 0,095 | 0,133 | 0,512 | 0,037 | 0,239 | 0,081 | 0,893 | 0,006 | 0,200 | 0,083 |

| **2-way ANOVA** | Liver MTTP  (au) | | Liver CPT1  (au) | | Fecal Triglycerides  (mg/g feces) | | IleaL MTTP  (au) | | Fecal bile acids  (mmol/g feces) | | Plasma bile acids  (μmol/L) | | |  |
| --- | --- | --- | --- | --- | --- | --- | --- | --- | --- | --- | --- | --- | --- | --- |
|  | p | η2 | p | η2 | p | η2 | p | η2 | p | η2 | p | η2 | | |
| M | 0,275 | 0,034 | 0,625 | 0,007 | 0,494 | 0,016 | 0,256 | 0,039 | 0,202 | 0,067 | 0,674 | | 0,005 |  |
| D | 0,013 | 0,219 | 0,032 | 0,174 | 0,011 | 0,258 | 0,005 | 0,277 | 0,532 | 0,051 | 0,091 | | 0,124 |  |
| MxD | 0,964 | 0,002 | 0,607 | 0,027 | 0,208 | 0,099 | 0,098 | 0,131 | 0,269 | 0,104 | 0,152 | | 0,100 |  |

| **2-way ANOVA** | Hepatic bile acids  (mmol/g protein) | | Ileal ASBT (au) | | Ileal IBABP  (au) | | Ileal OSTα  (au) | | Ileal OSTβ  (au) | | Ileal Proglucagon (au) | |
| --- | --- | --- | --- | --- | --- | --- | --- | --- | --- | --- | --- | --- |
|  | p | η2 | p | η2 | p | η2 | p | η2 | p | η2 | p | η2 |
| **M** | 0,265 | 0,036 | 0,696 | 0,004 | 0,969 | 0,000 | 0,595 | 0,008 | 0,140 | 0,061 | 0,953 | 0,000 |
| **D** | 0,003 | 0,288 | 0,242 | 0,076 | 0,237 | 0,077 | 0,047 | 0,161 | 0,032 | 0,179 | 0,023 | 0,223 |
| **MxD** | 0,082 | 0,137 | 0,019 | 0,198 | 0,077 | 0,133 | 0,323 | 0,062 | 0,215 | 0,084 | 0,240 | 0,091 |

| **2-way ANOVA** | Ileal PC 1/3 (au) | | Ileal DPP4  (au) | | Plasma GLP1 (pg/mL) | | Plasma FGF21 (pg/mL) | | Hepatic FGF21  (au) | | Plasma ANGII (pg/mL) | |
| --- | --- | --- | --- | --- | --- | --- | --- | --- | --- | --- | --- | --- |
|  | p | η2 | p | η2 | p | η2 | p | η2 | p | η2 | p | η2 |
| **M** | 0,825 | 0,002 | 0,848 | 0,001 | 0,004 | 0,210 | 0,516 | 0,013 | 0,716 | 0,004 | 0,009 | 0,181 |
| **D** | 0,098 | 0,131 | 0,003 | 0,328 | 0,628 | 0,026 | 0,000 | 0,547 | 0,000 | 0,433 | 0,002 | 0,300 |
| **MxD** | 0,206 | 0,091 | 0,094 | 0,150 | 0,921 | 0,005 | 0,136 | 0,114 | 0,491 | 0,039 | 0,505 | 0,038 |

| **2-way ANOVA** | Hepatic ACE (au) | | Ileal ACE  (au) | | Hepatic AVPR1A (au) | | Cardiac AVPR1A (au) | | Liver Glycogen (mg/g protein) | | Cardiac Glycogen (mg/g protein) | |
| --- | --- | --- | --- | --- | --- | --- | --- | --- | --- | --- | --- | --- |
|  | p | η2 | p | η2 | p | η2 | p | η2 | p | η2 | p | η2 |
| M | 0,394 | 0,020 | 0,367 | 0,023 | 0,696 | 0,004 | 0,976 | 0,000 | 0,973 | 0,000 | 0,522 | 0,012 |
| D | 0,563 | 0,031 | 0,000 | 0,490 | 0,000 | 0,385 | 0,481 | 0,043 | 0,391 | 0,051 | 0,001 | 0,349 |
| MxD | 0,459 | 0,042 | 0,147 | 0,101 | 0,428 | 0,046 | 0,002 | 0,320 | 0,397 | 0,050 | 0,347 | 0,059 |

| **2-way ANOVA** | Cardiac triglycerides  (mg/g protein) | | Liver triglycerides (mg/g protein) | | Ileal GPR41  (au) | | Ileal GPR43 (au) | | Ileal TGR5 (au) | | Hepatic MCT1 (au) | |
| --- | --- | --- | --- | --- | --- | --- | --- | --- | --- | --- | --- | --- |
|  | p | η2 | p | η2 | p | η2 | p | η2 | p | η2 | p | η2 |
| M | 0,813 | 0,002 | 0,557 | 0,011 | 0,017 | 0,149 | 0,148 | 0,061 | 0,101 | 0,077 | 0,029 | 0,132 |
| D | 0,019 | 0,209 | 0,483 | 0,044 | 0,018 | 0,201 | 0,027 | 0,191 | 0,005 | 0,266 | 0,486 | 0,042 |
| MxD | 0,584 | 0,031 | 0,352 | 0,063 | 0,182 | 0,090 | 0,411 | 0,051 | 0,375 | 0,056 | 0,163 | 0,101 |

| **2-way ANOVA** | Hepatic CD36 (au) | | Hepatic HDAC1 (au) | | Hepatic HDAC3 (au) | | Hepatic PDK4 (au) | | Hepatic CIDEC (au) | | Hepatic VLDLR (au) | |
| --- | --- | --- | --- | --- | --- | --- | --- | --- | --- | --- | --- | --- |
|  | p | η2 | p | η2 | p | η2 | p | η2 | p | η2 | p | η2 |
| M | 0,738 | 0,003 | 0,116 | 0,069 | 0,949 | 0,000 | 0,851 | 0,001 | 0,632 | 0,007 | 0,000 | 0,396 |
| D | 0,904 | 0,006 | 0,074 | 0,138 | 0,746 | 0,017 | 0,004 | 0,266 | 0,004 | 0,268 | 0,827 | 0,010 |
| MxD | 0,009 | 0,240 | 0,700 | 0,020 | 0,209 | 0,088 | 0,991 | 0,001 | 0,826 | 0,011 | 0,525 | 0,034 |

| **2-way ANOVA** | Cardiac SOD (mU/mg prot) | | Liver MDA (mmol/g tissue) | | Liver Catalase (mU/mg prot) | | Liver SOD (mU/mg prot) | | Cardiac MDA (mmol/g tissue) | | Cardiac Catalase (mU/mg prot) | |
| --- | --- | --- | --- | --- | --- | --- | --- | --- | --- | --- | --- | --- |
|  | p | η2 | p | η2 | p | η2 | p | η2 | p | η2 | p | η2 |
| M | 0,752 | 0,003 | 0,756 | 0,003 | 0,003 | 0,222 | 0,754 | 0,003 | 0,277 | 0,036 | 0,921 | 0,000 |
| D | 0,001 | 0,354 | 0,091 | 0,125 | 0,501 | 0,039 | 0,421 | 0,050 | 0,074 | 0,146 | 0,003 | 0,270 |
| MxD | 0,444 | 0,047 | 0,768 | 0,015 | 0,342 | 0,059 | 0,657 | 0,024 | 0,348 | 0,062 | 0,254 | 0,073 |
