# Supplementary material for: Tagatose consumption provokes metabolic syndrome features in rat males from mothers that consumed fructose during their pregnancy
Source: Mol Med. 2025 Dec 29;31:339. doi: 10.1186/s10020-025-01402-3 (PMC12751481; doi:10.1186/s10020-025-01402-3)
Supplement: Supplementary file 2 — Supplementary Material 2. [file 10020_2025_1402_MOESM2_ESM.docx]

**Supplemental Table 2**: Body weight (BW), and organ weight corrected by BW of control, fructose-, and tagatose of male progeny from control or fructose-fed mothers.

|  | **CONTROL MOTHERS** | | | | **FRUCTOSE MOTHERS** | | | |
| --- | --- | --- | --- | --- | --- | --- | --- | --- |
|  | **CONTROL** | **FRUCTOSE** | **TAGATOSE** | p | **CONTROL** | **FRUCTOSE** | **TAGATOSE** | p |
| **Body weight at day 0 (g)** | 412.75±6.73 | 414.4±10.51 | 446.16±7.14 |  | 363.93±13.83^##^ | 379.83±11.88^#^ | 376.13±7.98^###^ |  |
| **Body weight at day 21 (g)** | 457.90±10.26 | 463.93±11.65 | 480.04±9.26 |  | 418.53±18.51^#^ | 433.17±11.53 | 407.49±7.50^###^ |  |
| **Liver weight (g)/Body weight(g)** | 0.033±0.001 | 0.037±0.001 | 0.037±0.002 |  | 0.032±0.001 | 0.034±0.002 | 0.034±0.001 |  |
| **Heart weight (g)/Body weight(g)** | 0,0030±0,0001 | 0,0031±0,0000 | 0,0031±0,0000 |  | 0,0034±0,0002^#^ | 0,0032±0,0001 | 0,0033± 0,0001 |  |
| **WAT (g)/Body weight(g)** | 0.008±0.001 | 0.010±0.001 | 0.010± 0.001 |  | 0.013±0.002^##^ | 0.014±0.002^#^ | 0.010± 0.001 |  |
| **Body weight increase (g)** | 45.15±5.73 | 49.53±3.58 | 33.89±3.32 |  | 54.6±6.47 | 53.34±8.40 | 25.83±2.92 | **(FC vs FT)  **(FF vs FT) |

Body weight (BW) at the beginning (day 0) and the end (day 21) of nutritional treatments, and organ weight corrected by BW at day 21 of dietary treatment are shown. Data are means ± S.E. from 7-8 litters. Asterisks denote a significant difference (**, p < 0.01) between the groups with a different diet but the same mothers ́ diet. Hash symbols denote a significant difference (#, p < 0.05; ##, p < 0.01; ###, p < 0.001) as compared to the control mothers (groups with the same diet but different mother´s diet). There were no significant differences between groups with a different diet but the same mother´s diet. WAT: white adipose tissue.
