# Supplementary material for: Tagatose consumption provokes metabolic syndrome features in rat males from mothers that consumed fructose during their pregnancy
Source: Mol Med. 2025 Dec 29;31:339. doi: 10.1186/s10020-025-01402-3 (PMC12751481; doi:10.1186/s10020-025-01402-3)
Supplement: Supplementary file 3 — Supplementary Material 3. [file 10020_2025_1402_MOESM3_ESM.docx]

**Supplemental Table 3**: Ileal and hepatic gene (mRNA) expression of control (C), fructose- (F), and tagatose-supplemented (T) male progeny from control or fructose-fed mothers.

|  | **CONTROL MOTHERS** | | | | **FRUCTOSE MOTHERS** | | | |
| --- | --- | --- | --- | --- | --- | --- | --- | --- |
|  | **CONTROL** | **FRUCTOSE** | **TAGATOSE** | p | **CONTROL** | **FRUCTOSE** | **TAGATOSE** | p |
| **Ileal mRNA Gene Expression (a.u)** | | | | | | | | |
| ***Chrebp*** | 1.009 ±0.050 | 1.096 ±0.058 | 1.499 ±0.154 | ** CC vs CT  * CF vs CT | 1.272 ±0.130# | 1.204 ±0.012 | 1.469 ±0.065 |  |
| ***Fas*** | 0.915±0.078 | 1.329±0.0978 | 1.241±0.1401 |  | 0.863±0.039 | 0.866±0.136 ## | 1.513±0.1351 | ** FC vs FT  ** FF vs FT |
| ***Mttp*** | 1.028±0.02 | 1.211±0.022 | 1.144±0.086 |  | 0.862±0.038 | 1.041±0.121 | 1.264±0.072 | ** FC vs FT |
| ***Srebp1c*** | 1.019±0.076 | 1.187±0.097 | 1.081±0.075 |  | 0.973±0.049 | 1.035±0.136 | 1.113±0.116 |  |
| ***Ppara*** | 1.055 ±0.140 | 1.249 ±0.068 | 1.103 ±0.157 |  | 1.239 ±0.065 | 1.348 ±0.04004 | 1.193 ±0.094 |  |
| ***Acly*** | 1.004 ±0.036 | 1.047 ±0.059 | 1.179 ±0.082 |  | 0.968 ±0.061 | 0.997 ±0.095 | 1.267 ±0.081 | * FC vs FT  * FF vs FT |
| ***Acat2*** | 1.049 ±0.115 | 1.399 ±0.045 | 1.417 ±0.133 | * CC vs CT | 0.900 ±0.101 | 0.941 ±0.130 ## | 2.074±0.079 ### | *** FC vs FT  *** FF vs FT |
| ***Hmgr*** | 1.011±0.056 | 0.835±0.026 | 0.941±0.038 |  | 0.771±0.015 ## | 0.775±0.071 | 1.065±0.093 | ** FC vs FT  ** FF vs FT |
| ***Ldlr*** | 1.062±0.128 | 1.079±0.025 | 0.971±0.081 |  | 0.847±0.086 | 0.854±0.0709 | 1.223±0.130 | ** FC vs FT  ** FF vs FT |
| **Hepatic mRNA Gene Expression (a.u)** | | | | | | | | |
| ***Chrebp*** | 0.807±0.055 | 0.97±0.035 | 1.063±0.122 |  | 1.01±0.032 | 1.041±0.135 | 1.003±0.107 |  |
| ***Lpk*** | 1.053±0.120 | 2.249±0.191 | 1.632±0.125 | ***CC vs CF | 1.346±0.248 | 2.333±0.3428 | 1.236±0.045 | **FC vs FF  *FF vs FT |
| ***Fas*** | 16.36±2.81 | 43.87±7.44 | 34.67±5.76 | *CC vs CF | 25.85±5.16 | 43.91±10.92 | 34.29±7.48 |  |
| ***Dgat1*** | 1.030 ±0.095 | 1.069 ±0.069 | 1.132 ±0.126 |  | 0.684 ±0.04 ## | 1.196 ±0.053 | 0.835±0.036 # | ***FC vs FF  *FF vs FT |
| ***Dgat 2*** | 0.934±0.077 | 1.066±0.092 | 1.102±0.148 |  | 0.875±0.129 | 1.158±0.111 | 0.757±0.065 # | *FF vs FT |
| ***Srebp1c*** | 1.797±0.267 | 2.206±0.512 | 2.179±0.351 |  | 1.75±0.23 | 2.096±0.359 | 1.639±0.372 |  |
| ***Ppara*** | 1.010±0.051 | 0.981±0.076 | 0.926±0.070 |  | 1.159±0.094 | 1.179±0.111 | 1.054±0.120 |  |
| ***Pnpla3*** | 1.897±0.694 | 7.920±1.539 | 4.116±1.996 |  | 2.038±0.702 | 16.963±6.858 | 0.895±0.349 | **FC vs FF  **FF vs FT |
| ***Me*** | 1.173±0.228 | 3.713±0.404 | 2.421±0.522 | **CC vs CF | 0.973±0.131 | 4.020±0.8847 | 1.841±0.266 | ***FC vs FF  **FF vs FT |

Ileal and hepatic levels of specific mRNA genes are shown. Ileum and liver mRNA expression represents ChREBP signalling pathway genes related to lipogenesis; ileal and hepatic mRNA expression represents SREBP signalling pathway genes related to lipogenesis. Relative target gene mRNA levels were measured by Real Time PCR as explained in Materials and Methods, normalized to Rps29 levels and expressed in arbitrary units (a.u.). Data are means ± S.E. from 7 to 8 litters. Asterisks denote a significant difference (*, P < 0.05; **, P < 0.01) between the groups with a different diet but the same mothers ́ diet. Hash symbols denote a significant difference (#, P < 0.05; ##, P < 0.01) as compared to the control mothers (groups with the same diet but different mothers ́ diet). fructose. The first letter indicates whether the mothers had been supplied with tap water during pregnancy (C: control) or liquid fructose (F); and the second letter indicates the nutritional treatment without (C: control) or with additives, fructose (F) or tagatose (T), when they were adults. *ChREBP*: carbohydrate-responsive element-binding protein; and ChREBP signalling: *Lpk*: Liver pyruvate kinase; *Fas*: Fatty acid synthase; *Mttp*: microsomal triglyceride transfer protein; *Dgat*: diacylglycerol O-acyltransferase. *Ppara*: peroxisome proliferator-activated receptor alpha type. *Srebp1c*: sterol regulatory element-binding protein type 1c; and SREBP1c signalling: *Ldlr*: low-density lipoprotein receptor; *Acly*: ATP citrate lyase; *Me*: Malic enzyme; *Pnpla3*: patatin like domain 3, 1-acylglycerol-3-phosphate O-acyltransferase. Cholesterol synthesis pathway: *Acat2*: acetyl-CoA acetyl transferase type 2; *Hmgcr*: HMG-CoA reductase.
